# A new model calling procedure for Illumina BeadArray data

**DOI:** 10.1186/s12863-016-0398-x

**Published:** 2016-06-24

**Authors:** Gengxin Li

**Affiliations:** Department of Mathematics and Statistics, Wright State University, 3640 Colonel Glenn Hwy, Dayton, 45435 USA

**Keywords:** Dirichlet Process Gaussian mixture model, Gaussian mixture model, Genotype, HapMap, Single nucleotide polymorphism, Rare variants

## Abstract

**Background:**

Accurate genotype calling for high throughput Illumina data is an important step to extract more genetic information for a large scale genome wide association studies. Many popular calling algorithms use mixture models to infer genotypes of a large number of single nucleotide polymorphisms in a fast and efficient way. In practice, mixture models are mostly restricted to infer genotypes for common SNPs where their minor allele frequencies are quite large. However, it is still challenging to accurately genotype rare variants, especially for some rare variants where the boundaries of their genotypes are not clearly defined.

**Results:**

To further improve the call accuracy and the quality of genotypes on rare variants, a new model calling procedure, named M-D, is proposed to infer genotypes for the Illumina BeadArray data. In this calling procedure, a Gaussian Mixture Model and a Dirichlet Process Gaussian Mixture Model are integrated to infer genotypes.

**Conclusions:**

Applications to Illumina data illustrate that this new approach can improve calling performance compared to other popular genotyping algorithms.

## Background

Genome-wide association studies (GWAS) have been designed to discover many causal genetic variants contributing to human diseases [1, 2]. The success of GWAS relies heavily on the International HapMap Project where millions of single nucleotide polymorphisms (SNPs) have been widely identified on SNP arrays [3, 4]. With the rapid development in biotechnology, a leading producer, Illumina [5], is capable of offering SNP arrays with tremendously wide coverage of genetic variants in a fast and cost efficient way. A number of high dimensional intensity data are generated by this manufacturer, and various powerful genotyping algorithms are imperatively needed to accurately infer genotypes. Recently, several popular calling algorithms have been designed for Illumina platform, such as: BEAGLE with BEAGLECALL software [6], CRLMM [7, 8], GenCall [9], GenoSNP [10], and Iluminus [11]. In general, Illumina chip catalogs millions of SNPs and processes a large number of parallel samples, and the genotyping algorithms for the Illumina data is of the main interest.

With the application of single base extension (SBE) biochemistry technology [12], the Illumina data measures the pair of intensities with two alleles (*A* and *B*) at every SNP for each individual. Typically, a SNP with alleles *A* and *B* makes three possible genotype clusters, named *AA*, *AB*, and *BB*, and all possible genotypes of each SNP are called by various genotyping algorithms. One strategy is the population-based approach through which genotypes of all individuals within a SNP are inferred at one time, but its calling performances highly depend on the size of population. Thus, this method is not applicable for rare SNPs with low minor allele frequency (MAF). Another approach, GenoSNP, is designed to infer all SNP genotypes within one individual simultaneously, and is referred to as a SNP-based calling method. The applicability of this algorithm [10] relies on the assumptions that response features of all probes are similar. Compared to the population-based method, it would be unnecessary to collect a large number of samples for rare SNP calling due to the availability of high density SNPs. Unfortunately, this method leads to a larger proportion of SNPs breaking the Hardy-Weinberg (HW) principle which violates the assumption that commonly occurs in practice.

Most of the predominant calling algorithms employ the mixture models [13–15] to infer three genotype clusters. In particular, the mixture models developed from the population-based strategy work well for common SNPs but gradually lose their effectiveness for rare variants. To improve calculation accuracy, the mixture models need a sufficient number of observations in each genotype cluster to precisely estimate parameters. However, rare SNPs always contain a small number of individuals in one or two genotype clusters, and some rare SNPs with extremely small values of MAF may lose one or two clusters. This phenomenon creates two problems: (1) the number of components for rare SNPs is uncertain; (2) the boundaries of some genotype clusters are not clear for rare SNPs with sparsely populated observations. The problem about developing better inference for rare SNPs motivates the use of the Dirichlet Process (DP) Gaussian Mixture Model (GMM) [16–18]. One popular application of DP is clustering in the fields of brain imaging, information retrieval and genetics. To successfully perform a cognitive task, DP has been applied to analyze activation structures in functional magnetic resonance imaging [19]. DP has also been used to model relationships among documents in the field of information retrieval [20, 21]. For better understanding of ancestry history in the genetic study, DP was smoothly adopted to identify the sets of haplotypes corresponding to subpopulation [21]. Due to its good characteristics in clustering, this paper extends DP model to the genotyping area. Specifically, a DP prior plays a critical role in clustering data through defining a mixture model with a variable number of components. More importantly, its clustering and discreteness properties allows an easy partitioning of the data into different groups, even though some observations lack clear cluster membership. Besides, empirical studies have showed that GenoSNP can improve the genotyping quality for rare variants through calling a large number of SNPs within one individual. However, the genotype clusters implemented by GenoSNP may be in a shift away from their expected positions, which could result in many SNPs breaking the HW principle [5]. For a DP Gaussian Mixture Model (DP-GMM), its model selection procedure is based on a rich-gets-richer phenomenon [17], which indicates that the cluster with an extremely small number of observations is still toughly estimated. A reference SNP selection step [22] is incorporated here to infer genotypes of rare SNPs with extremely low MAF, and this new method may solve the HW principle problem.

In this paper, a new model calling procedure (M-D) is an approach that is made up of two models and one SNP selection procedure, namely Gaussian Mixture Model, DP Gaussian Mixture Model, and reference SNP selection. In brief, this method partitions SNPs into three groups in terms of the SNP’s MAF and the sample size of each cluster. In this method, three models are applied in three groups individually. The performance of M-D is evaluated through comparison with other genotyping algorithms for Illumina BeadArray data.

## Methods

### Illumina BeadArray data

The Illumina Omni BeadArray chip collects over one million SNPs per sample, and increasingly covers the newly identified variants. In the probe design, every beadtype that is capable of assaying two SNP alleles represents a SNP [12]. A large number of beadpools that include millions of beadtypes results in the ultimate production of the Illumina microarray. Here, Illumina data measures the pair of raw intensity at each beadtype for every sample, and the genotype clusters are estimated at this scale.

### Statistical models

#### Model I: Gaussian mixture model (GMM)

The pair of raw intensity **x**_*is*_=(*r*_*is*_,*g*_*is*_) for the *i*th individual at the *s*th SNP is the basic measurement. Within one SNP, all subjects’ intensity data may fall into three genotype clusters corresponding to three genotypes (AA, AB, BB) and one null component which collects the abnormal raw intensity measurements. Model I is a Gaussian Mixture Model [23] that is applied to the basic measurement **x**_*is*_. In principle, this model assigns each pair of raw intensities **x**_*is*_ to one of the components with probability *π*_*ks*_ for *k*= 1, 2 or 3. The relevant latent genotype class is measured by an indicator variable *z*_*is*_ generated from a multinomial distribution (*Mult*_3_) where *z*_*is*_= 1, 2 or 3. Then this Gaussian Mixture Model can be expressed as:

1 $$ \begin{aligned} &z_{is} \sim Mult_{3}(1,\mathbf{\pi}_{1s},\mathbf{\pi}_{2s},\mathbf{\pi}_{3s})\\ &\ell(\mathbf{x}_{s}|\boldsymbol{\Theta}_{s},\mathbf{z}_{s})=\prod_{i=1}^{n_{s}}\prod_{k=1}^{3}\Psi(\mathbf{x}_{is}|\boldsymbol{\mu}_{ks},\mathbf{\Sigma}_{ks})^{I(z_{is}=k)}\\ &{\mathbf{\mathit{i}} \boldsymbol{= 1,\ldots,} \mathit{n}_{\mathit{s}} \mathbf{, \mathit{s}} \boldsymbol{= 1,\ldots,} \mathbf{\mathit{S, k}} \boldsymbol{= 1, 2} \mathbf{\mathit{or}} \mathbf{3}} \end{aligned}  $$

where *n*_*s*_ is the total number of individuals observed at the *s*th SNP, and *S* is the total number of SNPs. *Ψ* denotes a normal density with mean ***μ***_*ks*_ and variance-covariance matrix **Σ**_*ks*_ in the *k*th component at the *s*th SNP; all pairs of raw intensity within the *s*th SNP are measured by **x**_*s*_=(**x**_1*s*_, **x**_2*s*_,..., $\mathbf {x}_{n_{s}s}$); the unknown parameters of the GMM is denoted by ***Θ***_*s*_=(***π***_*s*_, ***μ***_*s*_, **Σ**_*s*_) where ***π***_*s*_=(*π*_1*s*_, *π*_2*s*_, *π*_3*s*_), ***μ***_*s*_=(***μ***_1*s*_, ***μ***_2*s*_, ***μ***_3*s*_), and **Σ**_*s*_=(**Σ**_1*s*_, **Σ**_2*s*_, **Σ**_3*s*_).

The maximum likelihood estimates of the parameters are inferred [23]. For the indicator variable *z*_*is*_=*k*, the (*t*+1)th iteration is estimated by

2 $$ f_{k}\left(\mathbf{x}_{is};\boldsymbol{\Theta}_{s}^{t}\right)=\frac{\pi_{ks}^{t}\Psi \left(\mathbf{x}_{is};\boldsymbol{\mu}_{ks}^{t},\mathbf{\Sigma}_{ks}^{t}\right)} {\sum_{u=1}^{3}\pi_{us}^{t}\Psi\left(\mathbf{x}_{is};\boldsymbol{\mu}_{us}^{t},\mathbf{\Sigma}_{us}^{t}\right)}  $$

The iterative estimates of mean ***μ***_*ks*_ and variance-covariance matrix ***Σ***_*ks*_ are expressed as,

3 $$ \boldsymbol{\mu}_{ks}^{t+1}=\frac{\sum_{i=1}^{n_{s}} f_{k}\left(\mathbf{x}_{is};\boldsymbol{\Theta}_{s}^{t}\right)\mathbf{x}_{is}} {\sum_{i=1}^{n_{s}}f_{k}\left(\mathbf{x}_{is};\boldsymbol{\Theta}_{s}^{t}\right)}  $$

4 $$ \mathbf{\Sigma}_{ks}^{t+1}=\frac{\sum_{i=1}^{n_{s}} f_{k}\left(\mathbf{x}_{is};\boldsymbol{\Theta}_{s}^{t}\right) \left(\mathbf{x}_{is}-\boldsymbol{\mu}_{ks}^{t+1}\right) \left(\mathbf{x}_{is}-\boldsymbol{\mu}_{ks}^{t+1}\right)^{T}} {\sum_{i=1}^{n_{s}}f_{k}\left(\mathbf{x}_{is};\boldsymbol{\Theta}_{s}^{t}\right)}  $$

Two measurements Posterior Rate (PR: $p_{is}^{k}$) and the Average Posterior Rate (APR: *p*_*s*_) for the *s*th SNP are adopted to assess the quality of SNP calling [22]. Specifically, PR quantifies the strength of every individual’s cluster signal, and APR gives the average strength of all individuals at the *s*th SNP [22].

$$PR: p_{is}^{k}=\frac{P(\mathbf{x}_{is}|k)\pi_{ks}}{\sum_{u=1}^{3}P(\mathbf{x}_{is}|u)\pi_{us}} $$

$$APR: p_{s}=\frac{\sum_{k=1}^{3}\sum_{i=1}^{n_{ks}}p_{is}^{k}}{\sum_{k=1}^{3}n_{ks}} $$

Note that *P*(**x**_*is*_|*k*) is a conditional probability of the *i*th individual given that this subject is assigned to the *k*th cluster, and *n*_*ks*_ is the sample size of the *k*th cluster at the *s*th SNP.

#### Model II: Dirichlet Process Gaussian mixture model (DP-GMM)

Model I is a fast and efficient genotyping model for SNPs having large values of MAF. In real experiments, many SNPs with low MAF may result in the disappearance of one or two genotype clusters. Also even though some SNPs with low MAF display three genotype groups, some clusters may lack sufficient data to support and recognize. In this case, Model II, DP Gaussian Mixture Model, is motivated by the need to carry out the model selection for SNPs with an uncertain number of genotype clusters [24]. Generally speaking, this is a nonparametric Bayesian method that potentially allows a flexible number of mixture components and also provides estimates for the mixture component parameters and the relevant mixing proportions.

A DP Gaussian Mixture Model [24] fits the pair of raw intensity **x**_*is*_ into K-component Gaussian Mixture Model with K approaching a large number. The model is expressed as,

5 $$ \begin{aligned} &\ell(\mathbf{x}_{s}|\boldsymbol{\Theta}_{s},\mathbf{z}_{s})=\prod_{i=1}^{n_{s}}\prod_{k=1}^{K} \Psi(\mathbf{x}_{is}|\boldsymbol{\mu}_{ks},\mathbf{\Sigma}_{ks})^{I(z_{is}=k)}\\ &{\mathbf{\mathit{i}} \mathbf{= 1,\ldots,} n_{s} \mathbf{\mathit{, s}} \boldsymbol{= 1,\ldots,} \mathbf{\mathit{S}} \mathbf{\mathit{, k}} \boldsymbol{ = 1,\ldots,} \mathbf{\mathit{K}}} \end{aligned}  $$

where *K* is the total number of clusters. ***Θ***_*s*_=(***π***_*s*_, ***μ***_*s*_, **Σ**_*s*_) denotes the unknown parameters at the *s*th SNP where ***π***_*s*_=(*π*_1*s*_,..., *π*_*Ks*_), ***μ***_*s*_=(***μ***_1*s*_,..., ***μ***_*Ks*_), and **Σ**_*s*_=(**Σ**_1*s*_,..., **Σ**_*Ks*_). Generally, the number of observations within the *s*th SNP (*n*_*s*_) are partitioned into K components (*n*_1*s*_, *n*_2*s*_,..., *n*_*Ks*_) with relevant mixing proportions (*π*_1*s*_, *π*_2*s*_,..., *π*_*Ks*_). The distribution of *n*_1*s*_, *n*_2*s*_,..., *n*_*Ks*_ follows a multinomial distribution and its probability mass function is written by,

6 $$ {\small{\begin{aligned} {}p(n_{1s},n_{2s},\ldots n_{Ks}|\pi_{1s},\pi_{2s},\ldots,\pi_{Ks},n_{s})\,=\,\!\frac{n_{s}!}{n_{1s}!n_{2s}!\ldots n_{Ks}\,!}\!\prod_{k=1}^{K}\!\pi_{ks}^{n_{ks}} \end{aligned}}}  $$

where *n*_*s*_ = $\sum _{k=1}^{K}n_{ks}$ denotes the total number of individuals at the *s*th SNP. Then each pair of raw intensity for the *s*th SNP **x**_*is*_ has its own indicator *z*_*is*_ (i = 1,..., *n*_*s*_), and the distribution of indicator variables is expressed as,

7 $$ p(z_{1s},z_{2s},\ldots z_{n_{s}s}|\pi_{1s},\pi_{2s},\ldots,\pi_{Ks})=\prod_{k=1}^{K}\pi_{ks}^{n_{ks}}  $$

The model can then be expressed as:

8 $$ \begin{aligned} &\boldsymbol{\pi_{s}}|\alpha \sim Dir\left(\frac{\alpha}{K}, \frac{\alpha}{K},..., \frac{\alpha}{K}\right)\\ &z_{is}|\boldsymbol{\pi_{s}} \sim Discrete(\pi_{1s},\pi_{2s},...,\pi_{Ks}) \\ &\boldsymbol{R}_{ks}|\nu,\boldsymbol{S} \sim W\left(\nu, \boldsymbol{S}^{-1}\right) \\ &\boldsymbol{\mu}_{ks}|m,r,\boldsymbol{R}_{ks} \sim N(m, r\boldsymbol{R}_{ks}) \\ &\mathbf{x}_{is}|z_{is},\boldsymbol{\Theta}_{s} \sim N(\boldsymbol{\mu}_{z_{is}s},\mathbf{R}_{z_{is}s})\\ \end{aligned}  $$

where *α* is the DP concentration parameter and can be thought as the inverse variance of DP. The distribution of the reciprocal of *α* follows a Gamma distribution with 1 degree freedom and mean 1. K is the maximum number of clusters, then ***π***_***s***_ is distributed with a symmetric Dirichlet distribution with parameter $\frac {\alpha }{K}$. m and r are hyperparameters being the mean and relative precision of ***μ***_*ks*_, and the hyperparameters *ν* and ***S***^−1^ are degrees of freedom and inverse mean of **R**_*ks*_ where **R**_*ks*_ follows a Wishart distribution with parameters *ν* and ***S***^−1^, respectively.

The inference on Model II relies on the posterior distribution of each parameter conditional on all other parameters, then the parameters, hyperparameters and indicator variables are repeatedly sampled from their posterior distributions. In particular, the conditional posterior probabilities are proportional to the likelihood function multiplying priors. Then the posterior probabilities of the cluster indicator variable *z*_*is*_ conditional on all other variables are expressed as:

9 $${} \begin{aligned} &p(z_{is}=k|z_{-is},\boldsymbol{\mu}_{s}, \mathbf{R}_{s},\alpha,m,r,\nu,\mathbf{S}) \propto \\ &\left\{ \begin{array}{ll} \frac{n_{-i,ks}}{n_{s}-1+\alpha} N(\mathbf{x}_{is}|\boldsymbol{\mu}_{ks},\mathbf{R}_{ks}) \\ \quad \text{if }{k} \text{ is an existing cluster, and } n_{-i,ks} > 0\\ \frac{\alpha}{n_{s}-1+\alpha} \int p(\mathbf{x}_{is}|\boldsymbol{\mu}_{ks}, \mathbf{R}_{ks})p(\boldsymbol{\mu}_{ks}, \mathbf{R}_{ks}|m,r,\nu,\mathbf{S})d\boldsymbol{\mu}_{ks} d\mathbf{R}_{ks} \\ \quad \text{if k is a new cluster}\\ \end{array} \right. \end{aligned}  $$

Note that *p*(**x**_*is*_|***μ***_*ks*_,**R**_*ks*_) and *p*(***μ***_*ks*_,**R**_*ks*_|*m,r*,*ν*,**S**) are the likelihood function and the joint function of parameters (***μ***_*ks*_ and **R**_*ks*_), respectively. Once the optimal genotype clusters and their relevant component parameters are obtained, two measurements Posterior Rate (PR) and the Average Posterior Rate (APR) measuring the quality of the *s*th SNP can be calculated in the similar way.

#### Model III: Dirichlet Process Gaussian mixture model with reference SNP selection (DP-Ref)

A DP Gaussian Mixture Model with reference SNP selection step (DP-Ref) combines the benefits of the population-based method with the SNP-based approach. In this context, the reference SNP selection plays an important role in determining the effectiveness of Model III. A reference SNP is referred to as a good quality SNP providing sufficient information about three genotypes clusters, thus each SNP in the third group will be called with assistants of the carefully selected reference SNP. Practically, the final reference SNP is selected by a three-step procedure [22]. Through out this section, each SNP in the third group is denoted as the “T-SNP” that needs to be called with the support of a reference SNP, and the final reference SNP having good quality is defined as “R-SNP”

Step I. High MAF SNPs are selected as candidate reference SNPs. In fact, SNPs with large MAF (> 0.15) before the T-SNP are selected as R1-SNPs.

Step II. Good clustering property SNPs from R1-SNPs are further selected (denoted as R2-SNPs). This step requires three genotype clusters of R1-SNPs to contain at least 10 % of entire observations individually.

Step III. A SNP from R2-SNPs being remarkably similar to the T-SNP is selected (denoted as R-SNP). The resemblance between the T-SNP and each R2-SNP is measured by the cluster distance *D*_*t*_ [22]. For simplifying the calculation, two dimensional raw intensity vector **x**_*is*_ is projected to an univariate variable *y*_*is*_ [11], and the T-SNP and all R2-SNPs are classified into three genotype clusters in terms of this univariate variable.

$$y_{is}=\frac{r_{is}-g_{is}}{r_{is}+g_{is}} $$

10 $$ \left\{ \begin{array}{ll} y_{is} &\text{if } y_{is}< 0.5\\ y_{is} &\text{if } -0.5\leq y_{is}< 0.5 \\ y_{is} &\text{if } y_{is} \geq 0.5 \end{array} \right.  $$

Empirical studies show that the initial cutoffs dividing the univariate variable **y**_*s*_= ($\phantom {\dot {i}\!}y_{1s},...,y_{n_{s}s}$) ^*T*^ into three clusters can be fixed as 0.5 and −0.5. The cluster label of each individual would be roughly determined by the above equation.

We select one SNP from the third group as the T-SNP, then the cluster measure (*D*_*t*_) is to find the minimum distance between the T-SNP and R2-SNPs [22]. The SNP from R2-SNP gives the minimum distance will be the R-SNP. The cluster measure is calculated by,

11 $$ {{\begin{aligned} {}D_{t}=\underset{d; d\in \Xi}{min}\left\{\sum_{k=1}^{3}trace\left\{(\mathbf{x}_{kt}-\boldsymbol{\mu}_{kd}) ((\mathbf{\Sigma}_{kt}+\mathbf{\Sigma}_{kd})/2)^{-1}(\mathbf{x}_{kt}\,-\,\boldsymbol{\mu}_{kd})^{T} \right\}\right\} \end{aligned}}}  $$

Note that *Ξ* is the set of R2-SNPs selected for the T-SNP; **x**_*kt*_ and **Σ**_*kt*_ are the raw intensity vector and variance-covariance matrix in the *k*th cluster for the T-SNP; ***μ***_*kd*_ and **Σ**_*kd*_ are the mean and variance-covariance matrix of the *d*th R2-SNP; In brief, The final R-SNP will provide sufficient clusters information to assist the testing T-SNP.

A new augmented vector is generated by combining the T-SNP with the final reference SNP,

$$\begin{array}{*{20}l} \mathbf{m}_{t}= {\mathbf{x}_{t}\choose \mathbf{x}_{d}} \end{array} $$

where *d*∈*Ξ*, and the second model DP-GMM (Eqs. 6–9) will be applied to the combined raw intensities to identify the genotype clusters through the aid of the reference SNP.

## Application of new model (M-D)

This section focuses on the application of M-D. Specifically, entire SNPs are classified into three groups, and an appropriate model is selected to fit in each group. The classification standard relies on the calculations of MAF and the sample size of each genotype cluster through Model I. The reason for choosing this model is that GMM can quickly estimate the SNP’s MAF and the sample size of each genotype cluster. Other advanced models (Model II and III) will be applied to the selected SNPs with small MAFs. According to this calling procedure, any SNP will be classified by,

12 $$ \boldsymbol{x}_{s}=\left\{ \begin{array}{ll} \boldsymbol{x}_{s} \in g_{1} \text{if MAF } \geq 0.05.\\ \boldsymbol{x}_{s} \in g_{2} \text{if MAF } < 0.05 and b_{1} \leq n_{ks} < b_{2} \\ \quad \text{for any one of clusters.}\\ \boldsymbol{x}_{s} \in g_{3} \text{otherwise.}\\ \end{array}\right.  $$

Note that *n*_*ks*_ is the sample size of the *k*th cluster at the *s*th SNP. The first group (*g*_1_) collects SNPs with high MAF (≥ 0.05), and a large proportion of SNPs is in this group. The second group (*g*_2_) includes SNPs with low MAF (< 0.05) and a certain number of subjects in either existing genotype clusters. In this study, *b*_1_ and *b*_2_ are fixed as 3 and 10 to determine the number of SNPs recruited in *g*_2_. The last group (*g*_3_) collects the rest SNPs with low MAF and a small number of observations in one or two genotype clusters. In fact, SNPs in *g*_1_ can display three genotype clusters (one major homozygote, one minor homozygote and one heterozygote) with a large number of subjects in each cluster. The rest poor SNPs with low MAF are contained in *g*_2_ and *g*_3_ where some SNPs may not display three genotype clusters, either one or two clusters disappear and the existing cluster may contain very few observations. In particular, the classification between *g*_2_ and *g*_3_ is not fixed, and scientists can easily manage the allocation of SNPs between *g*_2_ and *g*_3_ through adjusting the values of *b*_1_ and *b*_2_.

The proposed new calling procedure is based on the partitions of SNPs.

13 $$ \left\{ \begin{array}{ll} g_{1}: \text{GMM } \\ g_{2}: \text{DP-GMM} \\ g_{3}: \text{DP-Ref}\\ \end{array}\right.  $$

In the first group, Model I (GMM) is applied to genotype SNPs. A sufficient number of observations are observed in three genotype clusters, which will greatly help the genotyping procedure identify the boundary of each cluster. In the second group, SNPs with low MAF, Model II (DP-GMM) can implement the model selection to search the appropriate number of clusters for each SNP, and DP’s clustering and discreteness properties assures the optimum partition of observations, even for a small number of observations in a genotype cluster. In the third group, the number of genotype clusters for each SNP is uncertain and an extremely small number of observations are observed in either one or two clusters. In this case, applying a DP-GMM alone for clustering is not enough due to a rich-gets-richer phenomenon [17] where the larger genotype cluster can greatly attract sparsely populated observations that originally belong to another cluster. In view of this situation, the reference SNP strategy [22] is applied to help DP-GMM call rare SNPs (DP-Ref). More importantly, the selection of models can be determined through adjusting *b*_1_ and *b*_2_ in Eq. 12. For example, when *b*_1_ takes a large value, a smaller proportion of rare SNPs may enter *g*_2_ and more rare SNPs are allocated to *g*_3_, thus GMM and DP-Ref will become major methods. If *b*_2_ takes a large value, a larger proportion of rare SNPs may be assigned to *g*_2_, then GMM and DP-GMM will become main methods. This flexible option provides more solutions for scientist who are interested in this genotyping method.

**Fig. 1:**
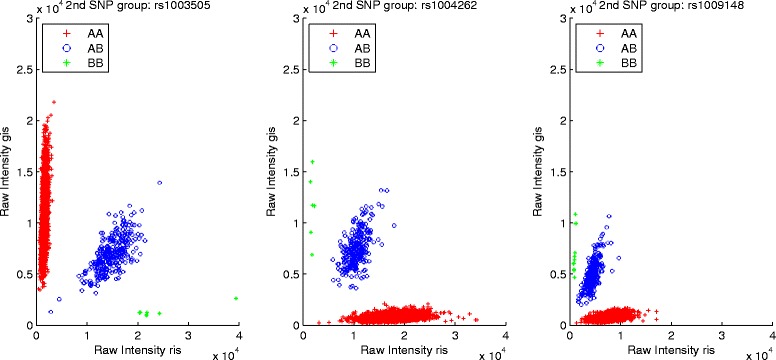
Performance of the DP Gaussian Mixture Model on genotyping three rare SNPs (rs1003505, rs1004262, rs1009148)

**Fig. 2:**
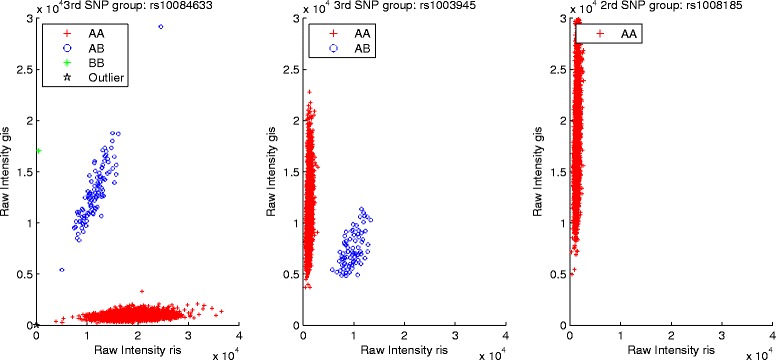
Performance of the DP Gaussian Mixture Model with reference SNP selection on genotyping three rare SNPs (rs10084633, rs1003945, rs1008185)

**Fig. 3:**
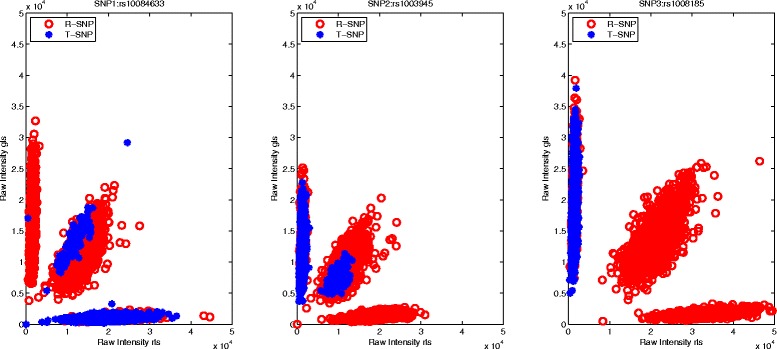
Performance of the reference SNP selection on genotyping three rare SNPs (rs10084633, rs1003945, rs1008185)

In this study, *b*_1_ and *b*_2_ are fixed as 3 and 10, then 88.6 % of SNPs are in *g*_1_, 4.03 % and 7.37 % of SNPs will be assigned to *g*_2_ and *g*_3_, respectively. More importantly, DP Gaussian Mixture Model is powerful to infer the cluster containing a certain number of observations, thus Fig. 1 displays the genotyping results of three SNPs inferred by DP-GMM (rs1003505 MAF: 0.0479, rs1004262 MAF: 0.0404, rs1009148 MAF: 0.0439). For the extremely rare variants in *g*_3_, DP-Ref is used to infer genotypes (rs10084633 MAF: 0.0166, rs1003945 MAF: 0.0118, rs1008185 MAF: 0), and the calling results are summarized in Fig. 2. To clearly illustrate the effect of the reference SNP on rare SNP calling in *g*_3_, Fig. 3 displays how the reference SNP help rare SNP be genotyped. It is clearly seen that our model could actively infer genotypes of rare SNPs under the support of the reference SNP.

## Results and discussion

### Illumina BeadArray data description

The proposed method M-D is applied to an Illumina data consisting of 1 million SNPs and 3258 samples. Specifically, there are 38 different HapMap samples [3] measured multiple times to produce 141 repeated HapMap samples in this data. SNP calls from the chromosome 22 are analyzed. The performance of *M*-*D* is compared to those of GenCall representing a population-based method and GenoSNP standing for a SNP-based approach. The compatible cutoffs of all three calling algorithms are carefully selected, such as: GenCall score (GC score ≥ 0.15) is used to filter good quality SNPs; GenoSNP and *M*-*D* collect good quality SNPs and samples through the posterior probability (≥ 85 %).

### Results

The performances of three calling algorithms are compared in terms of the call rate $\left (\frac {\text {{genotypes that can be inferred}}}{\text {{ genotypes that are supposed to be genotyped}}}\right)$ and the concordance rate measuring the genotype agreement between any two algorithms. The overall comparison results are given in Table 1. It is clearly seen that genotypes inferred from M-D, GenCall and GenoSNP are highly consistent, and genotypes from M-D are more consistent with those inferred from GenCall (99.93 %), than those from GenoSNP (99.65 %). This is because M-D is a population-based method in a wide sense, and the model selection of a DP and the reference SNP selection step in M-D greatly improve its call accuracy and call rate (Table 1).

**Table 1:** The comparisons of call rate and concordance rate among GenCall, GenoSNP and M-D

Algorithm 1	Algorithm 2	Call rate (%)		Concordance (%)
		Algorithm 1	Algorithm 2	
GenCall	M-D	96.71	99.71	99.93
GenoSNP	M-D	99.12	99.71	99.65
GenCall	GenoSNP	96.71	99.12	99.71

Note: The unit of Call Rate and Concordance Rate is percentage %; *M*-*D*: a new model calling procedure

Because most samples in this Illumina data are collected from the hospital, true genotypes of these sample are not known totally, so the high agreement among 3 algorithms can not tell us which method performs best. Fortunately, 141 HapMap samples are contained in this data, and the true genotypes of these HapMap samples are explored by the HapMap project as a gold standard. Table 2 provides the comparison results between each discussed method and a gold standard in terms of the call accuracy and the call rate. In brief, M-D gives the best call accuracy and the largest call rate, followed by GenoSNP and GenCall. For example: the largest call rate is achieved by M-D (99.78 %), followed by GenoSNP (99.14 %) and GenCall (96.79 %). Moreover, M-D offers the best call accuracy (99.44 %), followed by GenoSNP (98.52 %), and GenCall (96.63 %).

**Table 2:** The comparisons of call rates and accuracy on HapMap samples for overall SNPs

Criterion	Item	GenCall (%)	GenoSNP (%)	M-D (%)
All SNPs	Call rate	96.79	99.14	99.78
	Accuracy	96.63	98.52	99.44

Note: M-D: a new model calling procedure; Call rate: the percentage of valid genotypes; Accuracy: the percentage of consistent genotype between each calling method and the gold standard

Compared to the population-based methods (GenCall) and the SNP-based approaches (GenoSNP) [9, 10], the new model (M-D) is expected to perform better because it integrates a model selection step of DP and predominance of the population-based and the SNP-based strategies. In this study, SNPs are classified into 3 groups according to Eq. 12, and the comparison results corresponding to these 3 groups are summarized in Table 3. In brief, M-D gives the best call accuracy and largest call rate, followed by GenoSNP and GenCall. In particular, *g*_2_ and *g*_3_ collects whole rare SNPs, again, M-D still outperforms GenoSNP and GenCall on call accuracy and call rate.

**Table 3:** Comparisons of call rates and accuracy on HapMap samples for three SNP groups

Class	Prop	Item	GenCall	GenoSNP	M-D
*g*_1_	88.60 %	Call rate	96.59	99.13	99.77
		Accuracy	96.40	98.44	99.31
*g*_2_	4.03 %	Call rate	97.62	99.56	99.75
		Accuracy	97.53	99.45	99.59
*g*_3_	7.37 %	Call rate	96.60	99.14	99.70
		Accuracy	96.45	98.71	99.40

Note: M-D: a new model calling procedure; Call rate: the percentage of valid genotypes; Accuracy: the percentage of consistent genotype between each calling method and the gold standard; Class: indicates the three SNPs categories, such as: *g*_1_, *g*_2_ and *g*_3_; Prop: indicates the percentage of SNPs which belong to three groups, respectively

Hardy-Weinberg Equilibrium (HWE) test is another important criteria to examine the quality of SNPs. In this Illumina data, most samples are from four populations: Hispanic African-American, non-Hispanic African-American, Hispanic European-American, and non-Hispanic European-American. The HWE test (*P*-value < 0.0001) is applied to four populations separately. The total number of SNPs failing the HWE test are summarized in Table 4. A SNP-based method, GenoSNP, considers all SNPs calls within a sample at a time to improve genotyping quality for rare variants, but a large number of SNPs corresponding to four populations break the HW principle. In contrast, GenCall applies the population-based strategy to call all individuals within one SNP, so the calling results are less biased, and a small number of SNPs fail the HWE test. M-D is also a population-based model in a wide sense, and the quality of SNP calls is much better than that from GenoSNP, a moderate number of SNPs break the HW principle. In summary, M-D performs well on genotyping rare variants and controlling the quality of SNPs.

**Table 4:** Comparisons of Hardy-Weinberg Equilibrium test among GenCall, GenoSNP and M-D

Population	Num-Sample	Algorithm	# of failed SNPs
AA I	2005	GenCall	224
		GenoSNP	907
		M-D	422
AA II	83	GenCall	20
		GenoSNP	254
		M-D	80
EA I	867	GenCall	486
		GenoSNP	1024
		M-D	643
EA II	158	GenCall	40
		GenoSNP	348
		M-D	133

Note: AA I: African-Americans not of Hispanic Origin; AA II: African-Americans of Hispanic Origin; EA I: European Americans not of Hispanic Origin; EA II: European Americans of Hispanic Origin; Num-Sample: the number of subjects within each population; Algorithm: three algorithms in this table, that is, GenCall, GenoSNP, and M-D; # of failed SNPs: the number of SNPs fail the Hardy-Weinberg Equilibrium test within each population

### Discussion

The principle of a DP Gaussian Mixture Model is to run a model selection procedure to explicitly estimate the number of components for rare variants. The concentration parameter measures the inverse variance of DP, which suggests that a larger concentration parameter implies an increasing number of components [17]. It brings a new problem of how to select the appropriate strength of the prior to control the number of components. In particular, this parameter is sensitive to SNPs where sparsely populated observations are in one or two components. There might be better ways to define this parameter to help the DP Gaussian Mixture Model more efficiently call genotypes for rare variants.

The DP mixture model incorporates the reference SNP selection step to take advantage of the population-based strategy and the SNP-based strategy for improving the missing rate and call accuracy for rare SNPs. The successful application of M-D is also based on the selection of the reference SNP across the genome. In practice, it is difficult to search the reference SNP from the entire genome due to the heavy calculation burden. In these cases, the instrumental SNPs before the testing SNP are picked out. When some probes break the assumption about identical probe responses for various SNPs, searching the best reference SNP is still challenging. In particular, the method about accurately measures the similarity between the testing SNP and the reference SNP still needs to be improved.

## Conclusion

One classical genotyping approach is the population-based method, GenCall, and it requires a large number of observations to achieve a nice call accuracy. When the increasing number of rare variants are commonly identified on the large scale Illumina array, it is extremely difficult to successfully call genotypes for rare variants. A SNP-based method, GenoSNP, was designed to solve this challenging problem, but many more SNPs inferred from GenoSNP break the HW principle. In this paper, a new model calling procedure (M-D) is proposed to take benefits of a model selection step of a DP and the advantage of GenCall and GenoSNP to improve the quality of rare SNP calls. In brief, the new model calling procedure partitions SNPs into three classes in terms of MAF and the sample size of each cluster, and a DP Gaussian Mixture Model with or without reference SNP selection are applied to rare SNPs with low MAF. The finest performance of M-D is evaluated by comparing genotypes inferred by each discussed calling method to those from the HapMap project. Compared to GenCall and GenoSNP, M-D performs better on genotyping rare SNPs, and it also infers better quality of SNP calls than that from GenoSNP.

## Abbreviations

APR, average posterior rate; D _*t*_, cluster measure; DP, Dirichlet Process; DP-GMM, Dirichlet Process Gaussian mixture model; DP-Ref, Dirichlet Process Gaussian mixture model with reference SNP selection; GMM, Gaussian mixture model; GWAS, genome-wide association studies; HW, Hardy Weinberg; MAF, minor allele frequency; Multi_3_, a multinomial distribution; M-D, a new model calling procedure; PR, posterior rate; R-SNP, final reference SNP; SBE, single base extension; SNPs, single nucleotide polymorphisms; T-SNP, a SNP in the third group
